# Affinity Separation of Lectins Using Porous Membranes Immobilized with Glycopolymer Brushes Containing Mannose or *N*-Acetyl-d-Glucosamine

**DOI:** 10.3390/membranes3030169

**Published:** 2013-07-30

**Authors:** Yutaro Ogata, Hirokazu Seto, Tatsuya Murakami, Yu Hoshino, Yoshiko Miura

**Affiliations:** 1 Department of Chemical Engineering, Graduate School of Engineering, Kyushu University, 744 Motooka, Nishi-ku, Fukuoka 819-0395, Japan; E-Mails: y.ogata555@gmail.com (Y.O.); hirokazuseto@chem-eng.kyushu-u.ac.jp (H.S.); yhoshino@chem-eng.kyushu-u.ac.jp (Y.H.); 2 Center for Nano Materials and Technology, Japan Advanced Institute of Science and Technology, 1-1 Asahidai, Nomi, Ishikawa 923-1292, Japan; E-Mail: mtatsuya@jaist.ac.jp

**Keywords:** glycopolymer, polymer brush, atom transfer radical polymerization, lectin, affinity separation

## Abstract

Porous membranes with glycopolymer brushes were prepared as biomaterials for affinity separation. Glycopolymer brushes contained acrylic acid and D-mannose or *N*-acetyl-D-glucosamine, and were formed on substrates by surface-initiated atom transfer radical polymerization. The presence of glycopolymer brush was confirmed by X-ray photoelectron spectroscopy, contact angle, and ellipsometry measurements. The interaction between lectin and the glycopolymer immobilized on glass slides was confirmed using fluorescent-labeled proteins. Glycopolymer-immobilized surfaces exhibited specific adsorption of the corresponding lectin, compared with bovine serum albumin. Lectins were continuously rejected by the glycopolymer-immobilized membranes. When the protein solution was permeated through the glycopolymer-immobilized membrane, bovine serum albumin was not adsorbed on the membrane surface. In contrast, concanavalin A and wheat germ agglutinin were rejected by membranes incorporating D-mannose or *N*-acetyl-D-glucosamine, respectively. The amounts of adsorbed concanavalin A and wheat germ agglutinin was increased five- and two-fold that of adsorbed bovine serum albumin, respectively.

## 1. Introduction

Saccharides on the surface of cell membranes play a role in cell-cell, cell-extracellular matrix, and cell-virus interactions [1,2]. The interaction by monovalent saccharides is relatively weak, and can be amplified by multivalency, known as the glyco-cluster effect [3,4,5]. The multivalency of saccharides has been achieved by preparing glycopolymers, which are synthetic macromolecules containing saccharide side chains [6,7,8]. The application of biomaterials with the molecular recognition ability of glycopolymers has been reported in drug delivery systems, biosensors, and tissue engineering [9,10,11,12].

The chemical grafting of polymer layers on material surfaces to impart the polymer functionality has received much attention [13,14,15,16]. Surface-initiated atom transfer radical polymerization (SI-ATRP) allows high-densely polymer brushes to be prepared on various materials, with controlled molecular weight [17,18,19,20,21]. Reversible-deactivation radical polymerizations involving ATRP have been applied for synthesis of glycopolymer and preparation of biomaterial [22]. 

Size exclusion using fine porous membranes is often used for pathogen removal and separation, in which membranes based on target size are selected. Micro-filter and ultra-filter membranes are used to separate micrometer-sized bacteria and nanometer-sized proteins and viruses, respectively. Disadvantages of ultrafiltration membranes include the high pressure loss during solution permeation, the formation of caking layers, and low selectivity against similar-sized biomolecules. In contrast, the affinity adsorption system is more suitable for bioseparation because of its high selectivity. Some saccharides have specificity with pathogens such as toxic proteins, viruses, and bacteria [23,24,25,26]. When the glycopolymer is grafted on the membrane surface containing micro-scale pores, the target biomolecule is adsorbed because of convectional flow toward the saccharide ligand on the surface. Pressure loss and fouling by filtration residues are prevented by using micro-porous membranes.

In this study, glycopolymer brushes were grafted on glass slides and micro-porous membranes by SI-ATRP. Mannose (Man) and *N*-acetyl-D-glucosamine (GlcNAc) residues were incorporated into the polymer brushes. The specific interactions between glycopolymers containing Man and GlcNAc, and the lectins concanavalin A (Con A) and wheat germ agglutinin (WGA), respectively, were evaluated using fluorescence microarrays. Glycopolymer-immobilized membranes were used for the continuous separation of lectin.

## 2. Results and Discussion

### 2.1. Characterization of Poly(glyco-MA) and Poly(glyco-MA)-Immobilized Surfaces

**Figure 1:**
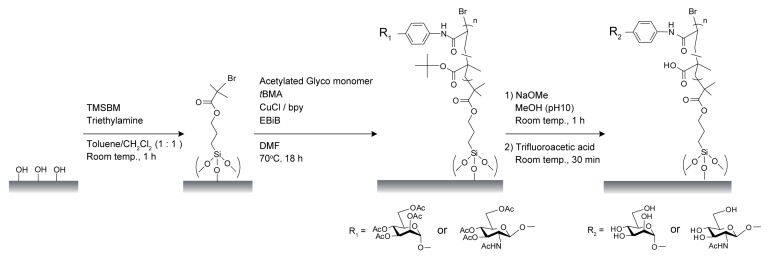
Preparation of poly(glyco-MA)-immobilized materials using surface-initiated atom transfer radical polymerization (SI-ATRP).

Poly(glyco-MA)-immobilized surfaces were prepared by SI-ATRP using glyco monomers and *t*BMA, as shown in Figure 1. Tsujii *et al*. reported that soluble polymers concurrently synthesized in solution media could be used to estimate the physical properties (molecular weight and distribution) of polymers grafted on surfaces [27]. In this study, EBiB as a soluble initiator was added to the reaction solution, and the resulting poly(glyco-MA) in solution was used for size exclusion chromatography (SEC) as an alternative to that immobilized on the surface (Figure S1 in Supplementary Information). The molecular weights and the molar mass dispersity (*Đ*_M_) are summarized in Table 1. The *Đ*_M_ values of poly(acetylated Man-*t*BMA) and poly(acetylated GlcNAc-*t*BMA) were higher than that of poly*t*BMA. The relatively high *Đ*_M_ values were attributable to copolymerization of different monomers, which had either methacrylate or acrylamide parts.

**Table 1:** Weight-average molecular weight (*M*_w_), number-average molecular weight (*M*_n_), and *Đ*_M_ of poly(glyco-MA) species synthesized in solution, determined by size exclusion chromatography (SEC).

Polymer	*M*_w_	*M*_n_	*Đ*_M_
Poly*t*BMA	24,800	17,100	1.4
Poly(acetylated Man-*t*BMA)	27,000	12,300	2.2
Poly(acetylated GlcNAc-*t*BMA)	14,700	9,500	1.5

**Figure 2:**
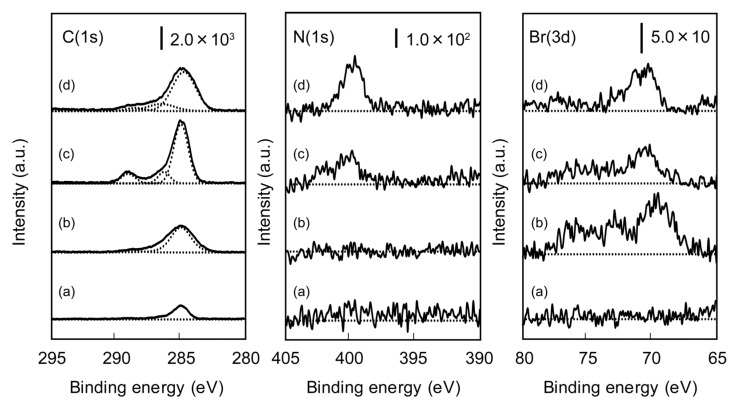
X-ray photoelectron spectroscopy (XPS) spectra of (**a**) unmodified; (**b**) TMSMA-immobilized; (**c**) poly(acetylated Man-*t*BMA)-immobilized; and (**d**) poly(acetylated GlcNAc-*t*BMA)-immobilized silicon wafers.

The presence of polymer brushes immobilized on the surface was confirmed by XPS measurements. XPS spectra of unmodified, TMSBM-immobilized, poly(acetylated Man-*t*BMA)-immobilized, and poly(acetylated GlcNAc-*t*BMA)-immobilized silicon wafers are shown in Figure 2. Compared with the C(1s) and Br(3d) spectra of unmodified and TMSBM-immobilized silicon wafers, new peaks were appeared after immobilization of TMSBM, suggesting that halogen-containing TMSBM was present on the surface. In the N(1s) spectrum of the TMSBM-immobilized silicon wafer, no peaks were observed because the initiator did not contain nitrogen atom. In the C(1s) and N(1s) spectra of the poly(acetylated glyco-*t*BMA)-immobilized silicon wafers, peaks corresponding to amide C–N, C=O, and N–C bonds were observed at 286.5, 288.1, and ~400 eV, respectively. The XPS data confirmed that polymer brushes were formed on the TMSBM-immobilized surface. Peaks were also observed in the Br(3d) spectra of the poly(acetylated glyco-*t*BMA)-immobilized silicon wafers, suggesting that bromide atom was retained at the terminal of polymer brush tips, consistent with the ATRP mechanism.

Contact angles of water droplets and thicknesses of layers on silicon wafers are shown in Table 2. The unmodified silicon wafer exhibited a low contact angle because of its high hydrophilicity, and the contact angle significantly increased once TMSBM was immobilized on the wafer. The TMSBM layer thickness was estimated by ellipsometry measurements to be 4.2 nm, suggesting that a multilayer of TMSBM was formed on the surface. The contact angle on poly(acetylated glyco-*t*BMA)-immobilized silicon wafers indicated a hydrophobic surface, which was consistent with polymer brushes containing numerous acetyl and *tert*-butyl groups. Total surface layer thicknesses were increased by *ca.* 9 nm upon forming poly(acetylated glyco-*t*BMA). The contact angles was slightly decreased after deprotection, suggesting that poly(acetylated Man-*t*BMA) and poly(acetylated GlcNAc-*t*BMA) immobilized on the surface were partially converted into poly(Man-MA) and poly(GlcNAc-MA), respectively. The relatively high contact angles on the poly(glyco-MA)-immobilized silicon wafers implied that the protecting groups were maintained on the surface. Thicknesses of the poly(glyco-MA)-immobilized surfaces were the same as those of the poly(acetylated glyco-*t*BMA)-immobilized surfaces. 

**Table 2:** Contact angles and polymer layer thicknesses of poly(glyco-MA)-immobilized silicon wafers.

Surface	Contact angle (°)	Thickness (nm)
Unmodified	7.5 ± 0.8	–
TMSBM-immobilized	76.5 ± 1.2	4.2
Poly(acetylated Man-*t*BMA)-immobilized	87.6 ± 5.0	14.1
Poly(Man-MA)-immobilized	60.4 ± 2.6	13.9
Poly(acetylated GlcNAc-*t*BMA)-immobilized	83.7 ± 3.0	12.3
Poly(GlcNAc-MA)-immobilized	69.3 ± 1.4	11.8

### 2.2. Microarrays of FITC-Protein Using Poly(glyco-MA)-Immobilized Glass Slides

**Figure 3:**
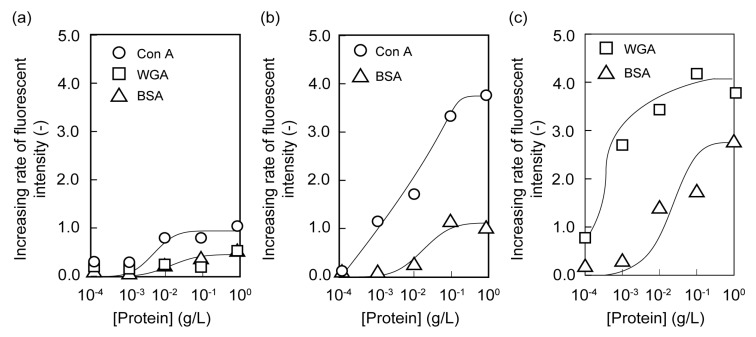
Increasing rate of fluorescence intensity for fluorescein isothiocyanate-labeled (FITC)-protein on (**a**) polyMA-immobilized; (**b**) poly(Man-MA)-immobilized; and (**c**) poly(GlcNAc-MA)-immobilized glass slides.

The biorecognition ability of poly(glyco-MA)-immobilized glass slides was evaluated using fluorescein isothiocyanate-labeled (FITC) proteins (Figures S2–S4). The increasing rates of fluorescent intensity for FITC-protein on polyMA-immobilized, poly(Man-MA)-immobilized, and poly(GlcNAc-MA)-immobilized glass slides at the various protein concentrations are shown in Figure 3. Protein adsorption was not observed on the polyMA-immobilized glass slide because of the electrostatic repulsion. FITC-Con A and FITC-WGA were adsorbed on poly(Man-MA)-immobilized and poly(GlcNAc-MA)-immobilized glass slides, respectively, at low FITC-protein concentrations. This suggests that the poly(glyco-MA) brushes were selectively bound to the corresponding lectin. 

**Figure 4:**
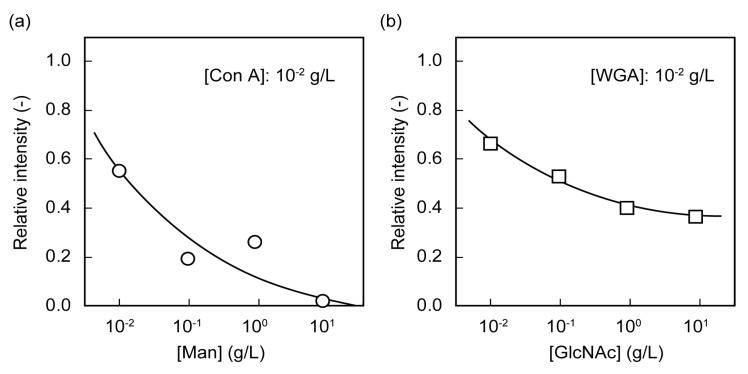
Relative fluorescence intensities of (**a**) Man-pretreated Con A on a poly(Man-MA)-immobilized glass slide and (**b**) GlcNAc-pretreated wheat germ agglutinin (WGA) on a poly(GlcNAc-MA)-immobilized glass slide, at various concentrations of saccharide.

To confirm the specificity of the poly(glyco-MA)-immobilized surface to lectin, Con A and WGA at concentrations of 1 × 10^−2^ g/L were complexed with Man and GlcNAc, respectively, prior to addition into wells on the glass slides. The relative intensities of Man-pretreated Con A on the poly(Man-MA)-immobilized glass slide and GlcNAc-pretreated WGA on the poly(GlcNAc-MA)-immobilized glass slide are shown in Figure 4. The relative intensities decreased with increasing saccharide concentration during lectin pretreatment. This decrease of relative intensity with increasing Man and GlcNAc concentrations indicated that the interactions of the Con A-Man unit in poly(Man-MA) and WGA-GlcNAc unit in poly(GlcNAc-MA) were specific.

### 2.3. Separation of Lectin Using Poly(glyco-MA)-Immobilized Membranes

**Figure 5:**
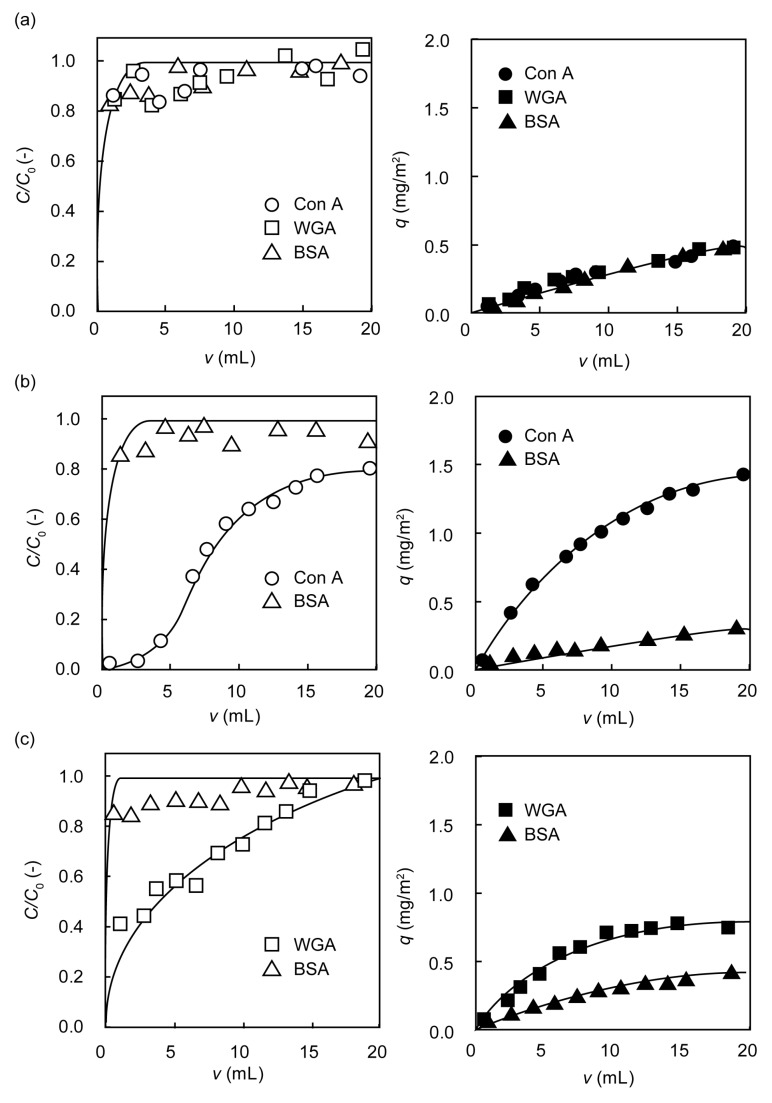
Breakthrough curves and amounts of protein adsorbed on (**a**) polyMA-immobilized; (**b**) poly(Man-MA)-immobilized; and (**c**) poly(GlcNAc-MA)-immobilized membranes.

Breakthrough curves and amounts of protein adsorbed during the permeation of protein solutions through the polyMA-immobilized, poly(Man-MA)-immobilized, and poly(GlcNAc-MA)-immobilized membranes are shown in Figure 5. When protein solutions were passed through the polyMA-immobilized membranes, breakthrough occurred just after the start of permeation. The amounts of protein adsorbed on the polyMA-immobilized membrane were <0.5 mg/m^2^. The adsorption behavior of poly(glyco-MA)-immobilized membranes was in good agreement with the microarray results, showing that Con A and WGA were dominantly adsorbed on the poly(Man-MA)-immobilized and poly(GlcNAc-MA)-immobilized surface, respectively. The amount of Con A adsorbed on the poly(Man-MA)-immobilized membrane was 1.5 mg/m^2^, and that of WGA on the poly(GlcNAc-MA)-immobilized membrane was 0.8 mg/m^2^.

The breakthrough point of Con A permeated through the poly(Man-MA)-immobilized membrane was ~5 mL, while most BSA was immediately passed through the poly(Man-MA)-immobilized membrane. The amount of Con A adsorbed on the poly(Man-MA)-immobilized membrane was five times that of BSA. No breakthrough point of WGA was observed for the poly(GlcNAc-MA)-immobilized membrane, and the amount of adsorbed WGA was twice that of adsorbed BSA. The micro-filter membrane prevented any size exclusion of protein. The passing of BSA through the poly(glyco-MA)-immobilized membranes indicated that the mechanism of lectin removal was not size exclusion but affinity adsorption. The poly(acetylated Man-*t*BMA)-immobilized membrane was obtusely adsorbed with Con A, and had no selectivity for protein due to hydrophobic interactions formed by numerous acetyl and *tert*-butyl groups (Figure S5). These results indicated that the poly(acetylated glyco-*t*BMA) was converted into the poly(glyco-MA). The poly(glyco-MA)-immobilized membranes containing micro-sized pores enabled selective bioseparation.

## 3. Experimental Section

### 3.1. Reagents and Materials

2-Bromoisobutyryl bromide, *tert*-butyl methacrylate (*t*BMA), and bovine serum albumin (BSA) were purchased from Sigma Co. (St. Louis, MO, USA). Trimethoxysilane (TMS), GlcNAc, tetradecyltrimethylammonium bromide, ethyl 2-bromo isobutyrate (EBiB) were purchased from Tokyo Chemical Industry Co. Ltd. (Tokyo, Japan). Triethylamine, *p*-nitrophenol, acryloyl chloride, 2,2′-bipyridyl (Bpy), ethylenediamine tetraacetic acid, and trifluoroacetic acid were purchased from Kanto Kagaku Co. Ltd. (Tokyo, Japan). Allyl alcohol, H_2_PtCl_6_∙H_2_O, CuCl, and sodium methoxide were purchased from Wako Pure Chemical Industries Ltd. (Osaka, Japan). Pd/C (10% Pd) was purchased from Merck & Co., Inc. (Rahway, NJ, USA). Con A, WGA, FITC-Con A, and FITC-WGA were purchased from J-Oil Mills Inc. (Tokyo, Japan). Glass slides (Matsunami Glass Ind. Ltd., Osaka, Japan) and SPG membranes (Lot No.: PEN08B05, SPG Technology Co., Ltd., Miyazaki, Japan) consisting of SiO_2_ and Al_2_O_3_ were used as matrices. The membrane had the effective length of 1.2 cm, average pore size of 1.9 µm, specific surface area of 1.1 m^2^/g, and weight of 0.1 g.

### 3.2. Synthesis

#### 3.2.1. 3-(Trimethoxysilyl)propyl 2-Bromo-2-methyl Propanoate (TMSBM)

Allyl alcohol (72 mmol, 1.2 equiv) and triethylamine (72 mmol, 1.2 equiv) were added dropwise to a stirred solution of 2-bromoisobutyryl bromide (60 mmol) in CH_2_Cl_2_ (300 mL). The reaction was monitored using thin layer chromatography (TLC), and upon completion the solution was concentrated by rotary evaporation. The crude product was purified by silica gel chromatography, using CHCl_3_ as the mobile phase. The solvent was removed, and allyl 2-bromo-2-methyl propanoate was obtained as a viscous liquid. The structure of product was confirmed using proton nuclear magnetic resonance (^1^H NMR) spectroscopy (JNM-ECP400, JEOL Ltd., Tokyo, Japan).

Allyl 2-bromo-2-methyl propanoate (42 mmol) was dissolved in anhydrous CH_2_Cl_2_ with N_2_ bubbling for 30 min. TMS (210 mmol, 5.0 equiv) and H_2_PtCl_6_∙H_2_O (44 mg) were added to the solution under a N_2_ atmosphere, with handling performed in a glove box. The solution was stirred under a N_2_ atmosphere at 40 °C overnight, with an attached condenser tube. Toluene was added and unreacted TMS was removed by rotary evaporation. TMSBM was obtained as a toluene solution.

**Allyl 2-bromo-2-methyl propanoate** (4.8 g, 70%); ^1^H NMR (400 MHz, CDCl_3_): *δ* 5.86 (1 H, ddd, –CH=CH_2_), *δ* 5.33 (1 H, d, –CH=CH_2_), *δ* 5.23 (1 H, s, –CH=CH_2_), *δ* 4.60 (2 H, m, –O–CH_2_–CH = CH_2_), *δ* 1.78 (3 H, s, CH_3_–C), TLC (EtOAc:hexane = 1:30): *R*_f_ = 0.60.

#### 3.2.2. Glyco Monomers

*p*-Acrylamidophenyl 2,3,4,6-tetra-*O*-acetyl-α-D-mannopyranoside (acetylated Man monomer) was synthesized as described previously [28]. *p*-Acrylamidophenyl 3,4,6-tri-*O*-acetyl *N*-acetyl-glucosamine (acetylated GlcNAc monomer) was synthesized similarly to the acetylated Man monomer. Specifically, GlcNAc (22.6 mmol) was dissolved in acetyl chloride (25 mL), and the solution was stirred for 1 day. After reaction completion was confirmed using TLC, cooled water and ethyl acetate were added, and then the product was extracted into the organic layer. The organic layer was washed once with saturated aqueous NaHCO_3_, twice with saturated aqueous NaCl, and twice with water, then dried over MgSO_4_ and filtered. The solvent was evaporated, and the residue was purified by flash chromatography with ethyl acetate to afford chloro 2-acetamido-3,4,6-tri-*O*-acetyl-2-deoxy-α-D-glucopyranoside. The structure of the product was confirmed using ^1^H NMR.

A solution of chloro 2-acetamido-3,4,6-tri-*O*-acetyl-2-deoxy-α-D-glucopyranoside (12.1 mmol) in dichloromethane (30 mL) was mixed with tetradecyltrimethyl ammonium bromide (12.1 mmol, 1.0 equiv) and *p*-nitrophenol (24.2 mmol, 2.0 equiv) in 1 mol/L aqueous NaOH (30 mL), and the solution was stirred. After reaction completion was confirmed using TLC, the product was extracted three times with chloroform. The organic layer was washed three times with 1 mol/L aqueous NaOH, twice with saturated aqueous NaCl, and twice with water, then dried over MgSO_4_ and filtered. The solvent was evaporated, and the residue was purified by gradient flash chromatography with chloroform and chloroform/methanol (10:1) to afford *p*-nitrophenyl 2-acetamido-3,4,6-tri-*O*-acetyl-2-deoxy-β-D-glucopyranoside. The structure of product was confirmed using ^1^H NMR.

Pd/C (96 mg, 5 wt %) was added to a solution of *p*-nitrophenyl 2-acetamido-3,4,6-tri-*O*-acetyl-2-deoxy-β-D-glucopyranoside (4.8 mmol) in hydrous methanol. The reaction was stirred at room temperature under a hydrogen atmosphere for 2 h. The reaction completion was confirmed using TLC. The solution was filtered through glass fiber paper, and then concentrated *in vacuo*. The residue was dissolved in DMF (30 mL), and acryloyl chloride (5.3 mmol, 1.3 equiv) and triethylamine (5.3 mmol, 1.3 equiv) were added at 0 °C. The solution was stirred at room temperature for 2 h, and the reaction was monitored using TLC. The solution was concentrated *in vacuo*, dissolved in chloroform, washed twice with saturated aqueous NaHCO_3_, twice with saturated aqueous NaCl, and twice with water, and dried over MgSO_4_ and filtered. The solvent was evaporated, and the residue was purified by flash chromatography with chloroform/methanol (10:1) to afford the acetylated GlcNAc monomer. The structure of product was confirmed using ^1^H NMR.

**Chloro 2-acetamido-3,4,6-tri-*O*-acetyl-2-deoxy-α-D-glucopyranoside** (2.6 g, 31%); ^1^H NMR (400 MHz, CDCl_3_): *δ* 6.17 (1 H, d, *J*_1,2_ 3.6 Hz, H-1), 5.80 (1 H, d, *J*_–NH_ 8.7 Hz, –NH), 5.30 (1 H, dd, *J*_2,3_ 20.1 Hz, *J*_3,4_ 9.6 Hz, H-3), 5.20 (1 H, t, *J*_3,4_ = *J*_4,5_ 9.6 Hz, H-4), 5.20 (1 H, dd, *J*_2,3_ 11.4 Hz, *J*_3,4_ 3.3 Hz, H-3), 4.51 (1 H, ddd, *J* 10.8 Hz, 8.7 Hz, 3.9 Hz, H-5), 4.30–4.22 (2 H, m, H-6a, H-6b), 4.14–4.08 (1 H, m, H-2), 4.04 (1 H, dd, *J*_6a,6b_ 11.1 Hz, *J*_5,6b_ 6.6 Hz, H-6b), 2.09 (3 H, s, –OAc), 2.01 (3 H, s, –OAc), 2.01 (3 H, s, –OAc), 1.97 (3 H, s, –NHAc), TLC (EtOAc:hexane = 3:1): *R*_f_ = 0.65.

***p*-Nitrophenyl 2-acetamido-3,4,6-tri-*O*-acetyl-2-deoxy-β-D-glucopyranoside** (2.9 g, 50%); *δ* 8.17 (2 H, m), 7.05 (2 H, m), 5.69 (1 H, d, *J*_–NH,2_ 8.7 Hz, –NH), 5.44 (1 H, d, *J*_1,2_ 8.3 Hz, H-1), 5.43 (1 H, dd, *J*_2,3_ 10.5 Hz, *J*_3,4_ 9.7 Hz, H-3), 5.12 (1 H, t, *J*_3,4_ 9.7 Hz, H-4), 4.26 (1 H, dd, *J*_6a,6b_ 12.3 Hz, *J*_5,6a_ 5.5 Hz, H-6a), 4.15 (1 H, dd, *J*_6a,6b_ 12.3 Hz, *J*_5,6b_ 2.4 Hz, H-6b), 4.10 (1 H, ddd, *J*_2,3_ 10.5 Hz, *J*_–NH_ 8.7 Hz, *J*_1,2_ 8.3 Hz, H-2), 4.00 (1 H, ddd, *J*_4,5_ 9.7 Hz, *J*_5,6a_ 5.5 Hz, *J*_5,6b_ 2.4 Hz, H-5), 2.15 (3 H, s, –OAc), 2.06 (3 H, s, –OAc), 2.04 (3 H, s, –OAc), 1.94 (3 H, s, –NHAc), TLC (chloroform:methanol = 10:1): *R*_f_ = 0.50.

***p*-Acrylamidophenyl 3,4,6-tri-*O*-acetyl *N*-acetyl-glucosamine** (0.56 g, 30%); *δ* 10.28 (1 H, 2, –NH–), 8.18–8.15 (2 H, d, –NHAc), 7.74–7.71 (2 H, d, –Ph), 7.06–7.04 (2 H, d, –Ph), 6.53–6.50 (1 H, m, CH=CH_2_), 6.34–6.30 (1 H, m, CH=CH_2_), 5.74–5.73 (1 H, m, CH=CH_2_), 5.46–5.44 (1 H, d, H-1), 5.44–5.38 (1 H, t, H-3), 5.07–5.02 (1 H, t, H-4), 4.34–4.30 (1 H, d, H-2), 4.21–4.16 (3H, m, H-5, H-4), 2.05–1.88 (12 H, d, –OAc), TLC (chloroform:methanol = 10:1): *R*_f_ = 0.37.

#### 3.2.3. FITC-BSA

FITC-BSA was synthesized by incubating 100 mg of BSA in NaOH solution (1 mmol/L, 10 mL) with fluorescein isothiocyanate isomer (3 µmol, Dojindo Laboratories, Kumamoto, Japan) for 3 h, and subsequently purifying with a dialysis membrane (molecular weight cut off: 1000 Da, Spectra/Por, Spectrum Laboratories Inc., Rancho Dominguez, CA, USA) against water. When the obtained FITC-BSA solution (1 g/L) was excited at 480 nm, the peak with a fluorescence intensity of 97 was observed at 530 nm in emission spectrum.

### 3.3. Preparation of Initiator-Immobilized Glass Slides and Membranes for SI-ATRP

TMSBM as the SI-ATRP initiator was immobilized on silicon wafers, glass slides, and membranes. Silicon wafers and glass slides were each sequentially sonicated in acetone, ethanol, and water, and then cleaned by UV/O_3_ treatment for 30 min. The membrane was washed with piranha solution (sulfuric acid:hydrogen peroxide = 3:1) for 30 min. The silicon wafers, glass slides, and membranes were immersed in TMSBM solution (0.12 mmol/L, toluene:CH_2_Cl_2_ = 1:1, 2.5 mL) with triethylamine (500 µL), and were then incubated at room temperature for 1 h. The resulting substrates were washed with CH_2_Cl_2_ and methanol, and were dried to obtain the TMSBM-immobilized surfaces.

### 3.4. Surface Modification of Glass Slides and Membranes with Glyco-Polymer Brushes via SI-ATRP

Polymer brushes with glyco residues were concurrently produced in solution and on the surfaces of the glass slide, silicon wafer, and membrane, by SI-ATRP in a flask. The glyco monomer (0.4 mmol), *t*BMA (3.6 mmol), and Bpy (20 µmol) were dissolved in anhydrous DMF (3 mL), and the solution was degassed using N_2_. EBiB (40 µmol) in anhydrous DMF (1 mL) was added to the solution using a syringe, and the solution was degassed using N_2_. The TMSBM-immobilized substrate and CuCl (10 µmol) were transferred to a flask under a N_2_ atmosphere. The monomer-containing solution was introduced to the flask using a double-headed needle. The solution mixture was deaerated by freeze-thaw cycling, and then the substrate was incubated in the solution at 70 °C for 18 h. Air was introduced to the solution to stop the reaction, and the resulting substrate was washed with aqueous ethylenediamine tetraacetic acid (0.25 mmol/L) and chloroform. The substrate was immersed in sodium methoxide in methanol (pH: 10) at room temperature for 1 h to deprotect acetyl group within glyco units. The substrates were subsequently immersed in trifluoroacetic acid at room temperature for 30 min to *tert*-butyl groups from methacrylate (MA) units. Thus, poly(glyco-MA)-immobilized glass slides and membranes were obtained. PolyMA brushes on substrates were also prepared for adsorption reference. The surficial properties of poly(glyco-MA)-immobilized silicon wafers were evaluated by X-ray photoelectron spectroscopy (XPS, AXIS-ultra, Shimadzu/Kratos, Kyoto, Japan), contact angle (DropMaster 300, Kyowa Interface Science, Saitama, Japan), and ellipsometry (NL-MIE, Nippon Laser & Electronics Lab., Nagoya, Japan) measurements.

### 3.5. SEC Determination

The molecular weights of soluble copolymers, produced in solution media, were determined using SEC. After ATRP, the solution was concentrated by vacuum drying. The unreactive monomer, initiator, and catalyst were removed by dialysis (molecular weight cut off: 3.5 kDa) against DMSO for 3 days. DMSO was removed *in vacuo*, and then the residue was dissolved in THF to prepare the sample solution (10 g/L). The molecular weights were determined using SEC with a column (Shodex LF804 column, Showa Denko KK., Kanagawa, Japan) connected to a degasser (DG-980-50, JASCO Co., Tokyo, Japan), pump (PU-2080 Plus, JASCO Co., Tokyo, Japan), UV detector (UV970, JASCO Co., Tokyo, Japan), and refractive index detector (RI-2031 Plus, JASCO Co., Tokyo, Japan). The mobile phase was THF permeating at a flow rate of 0.5 mL/min. The calibration curve was prepared using polystyrene standards (Showa Denko K. K., Tokyo, Japan) as first-order approximation between logarithmic molecular weight and retention time.

### 3.6. Adsorption of Fluorescent-Labeled Lectin on the Surface of Poly(glyco-MA)-Immobilized Glass Slide

To evaluate the protein recognition ability, the fluorescence images of the poly(glyco-MA)-immobilized surface were observed using FITC-labeled proteins. Con A is a Man-binding lectin, and WGA is a GlcNAc-binding lectin. Silicon rubber wells (diameter: 3 mm) were attached to poly(glyco-MA)-immobilized glass slides, and FITC-protein solutions with the various concentrations in phosphate-buffered saline (pH: 7.4, 10 µL) were added into the wells. After incubation in the dark for 2 h followed by washing with the buffer, surfaces adsorbed with FITC-proteins were observed using a fluorescence microscope (BZ-8000, Keyence Corporation, Osaka, Japan). The increasing rate of fluorescent intensity on surfaces adsorbed with FITC-protein was estimated from the average gray value obtained by ImageJ software, according to:

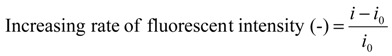
(1)
where *i*_0_ and *i* are the average gray values of the surface incubated with the buffer and the surface incubated with FITC-protein solution, respectively.

To confirm the specificity of poly(glyco-MA)-immobilized surfaces to the lectins, Man and GlcNAc were added into FITC-Con A and FITC-WGA solutions (10^−2^ g/L), respectively. These solutions were incubated to prepare saccharide-pretreated lectins. Solutions of saccharide-pretreated lectin with various saccharide concentrations were added into the wells on poly(Man-MA)-immobilized and poly(GlcNAc-MA)-immobilized glass slides. After incubation in the dark for 2 h and washing with the buffer, surfaces adsorbed with FITC-proteins were observed using a fluorescence microscope. The relative fluorescent intensity on the surface adsorbed with FITC-protein in the presence of saccharide against that in the absence of saccharide was estimated from average gray values.

### 3.7. Rejection of Lectin on the Surface of Poly(glyco-MA)-Immobilized Membrane

The lectins were continuously rejected using the poly(glyco-MA)-immobilized membranes. A feed solution of each protein (10 mg/L) was passed through the polyMA-immobilized, poly(Man-MA)-immobilized, and poly(GlcNAc-MA)-immobilized membranes at 10 mL/h. The effluent was continuously collected, and the concentration of protein in the effluent was determined using the Bradford method [29]. The amount of protein adsorbed on the membrane (*q*) was estimated from:

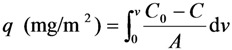
(2)
where *C*_0_, *C*, *A*, and *v* are the concentration of protein in the feed solution, concentration of protein in the effluent solution, the surface area of membrane, and volume of effluent solution, respectively. The feed solution of each protein was also passed through the poly(acetylated Man-*t*BMA)-immobilized membrane.

## 4. Conclusions

Poly(glyco-MA) polymer brushes were formed on silicon wafers, glass slides, and porous membranes by SI-ATRP as a grafting method. Poly(glyco-MA)-immobilized surfaces exhibited a high recognition ability for lectin, corresponding to the saccharide spices within the polymer brush. The saccharide specificity has considerable potential for the removal of small pathogens, such as toxic proteins and viruses. Glycopolymer-immobilized porous materials are applicable in medical treatment and environmental detoxification.

## Supplementary Files

Supplementary File 1 Supplementary (PDF, 4025 KB)
