# Supplementary material for: Affinity Separation of Lectins Using Porous Membranes Immobilized with Glycopolymer Brushes Containing Mannose or N-Acetyl-d-Glucosamine
Source: Membranes (Basel). 2013 Jul 30;3(3):169–81. doi: 10.3390/membranes3030169 (PMC4021937; doi:10.3390/membranes3030169)

Supplementary Information

Figure S1. Size exclusion chromatograms of poly*t*BMA, poly(acetylated Man-*t*BMA), and poly(acetylated GlcNAc-*t*BMA) synthesized in solution. Refractive index detector and UV detector were used for poly*t*BMA and poly(acetylated glyco-*t*BMA), respectively. The mobile phase was THF permeating at a flow rate of 0.5 mL/min. Peak in the chromatogram of the poly(acetylated Man-*t*BMA) was overlapped with a ghost peak. The peak was divided by Peak Fitting software, and the M_w, M_n, and $Đ_M$ of the poly(acetylated Man-*t*BMA) were estimated from the divided peak.

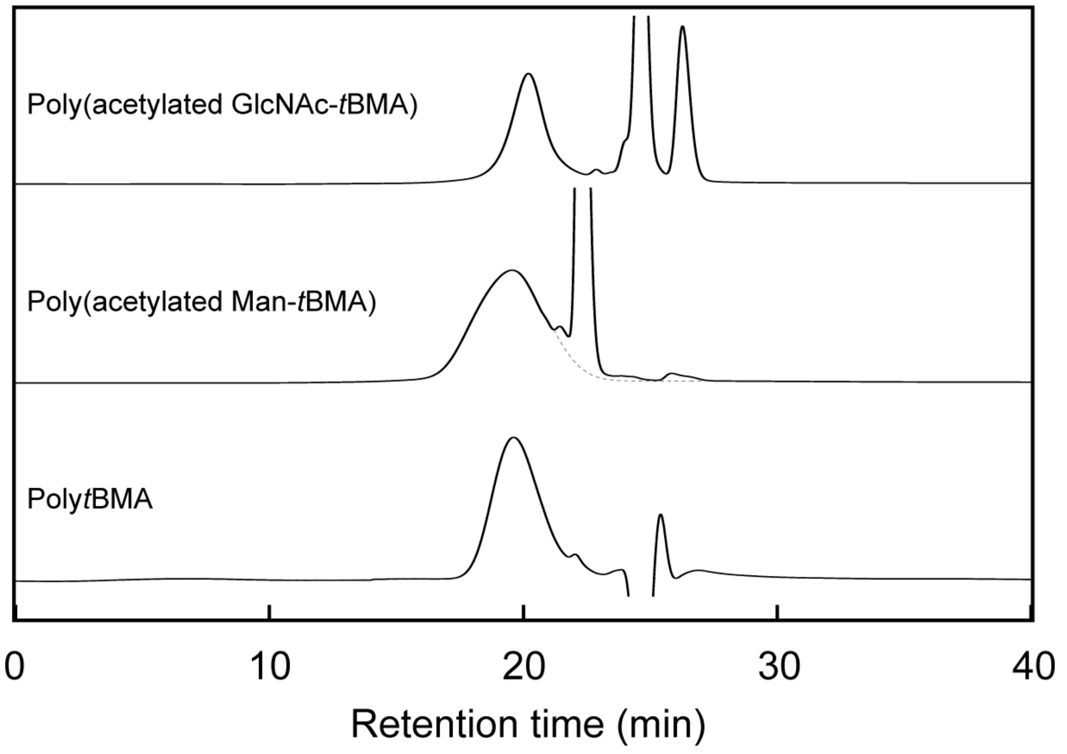

Figure S2. Fluorescent images on polyMA-immobilized glass slide adsorbed with (**a**) FITC-Con A; (**b**) FITC-WGA; and (**c**) FITC-BSA.

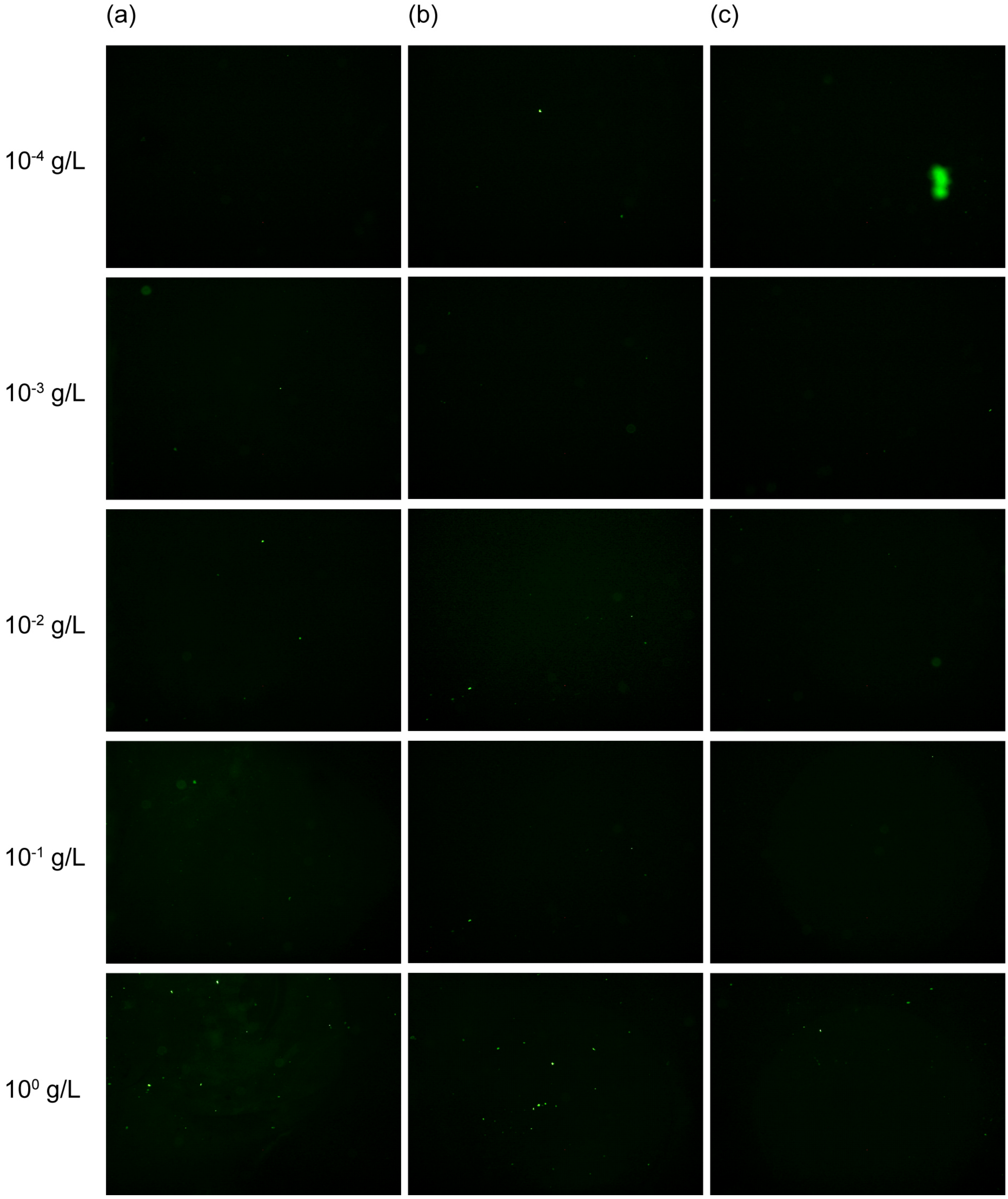

Figure S3. Fluorescent images on poly(Man-MA)-immobilized glass slide adsorbed with (**a**) FITC-Con A and (**b**) FITC-BSA.

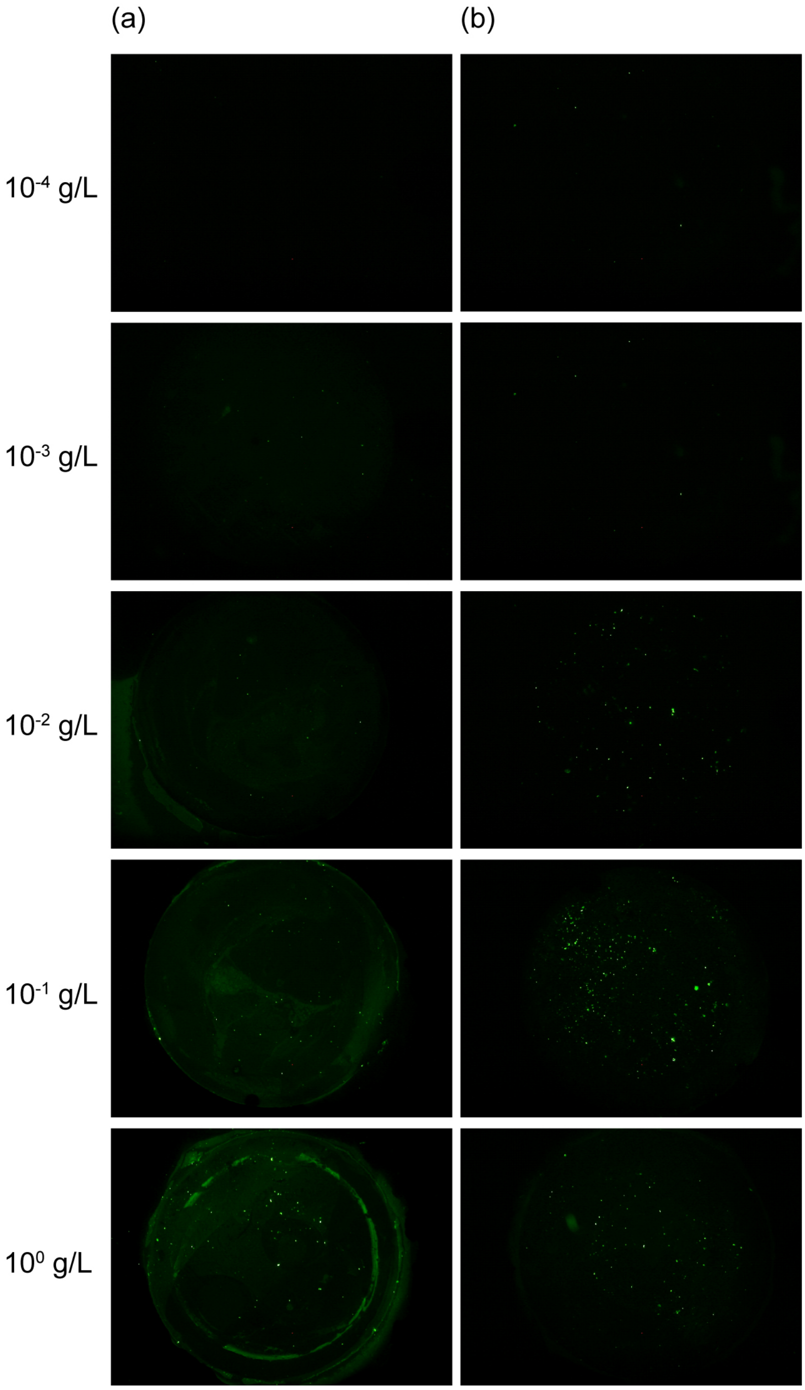

Figure S4. Fluorescent images on poly(GlcNAc-MA)-immobilized glass slide adsorbed with (**a**) FITC-WGA and (**b**) FITC-BSA.

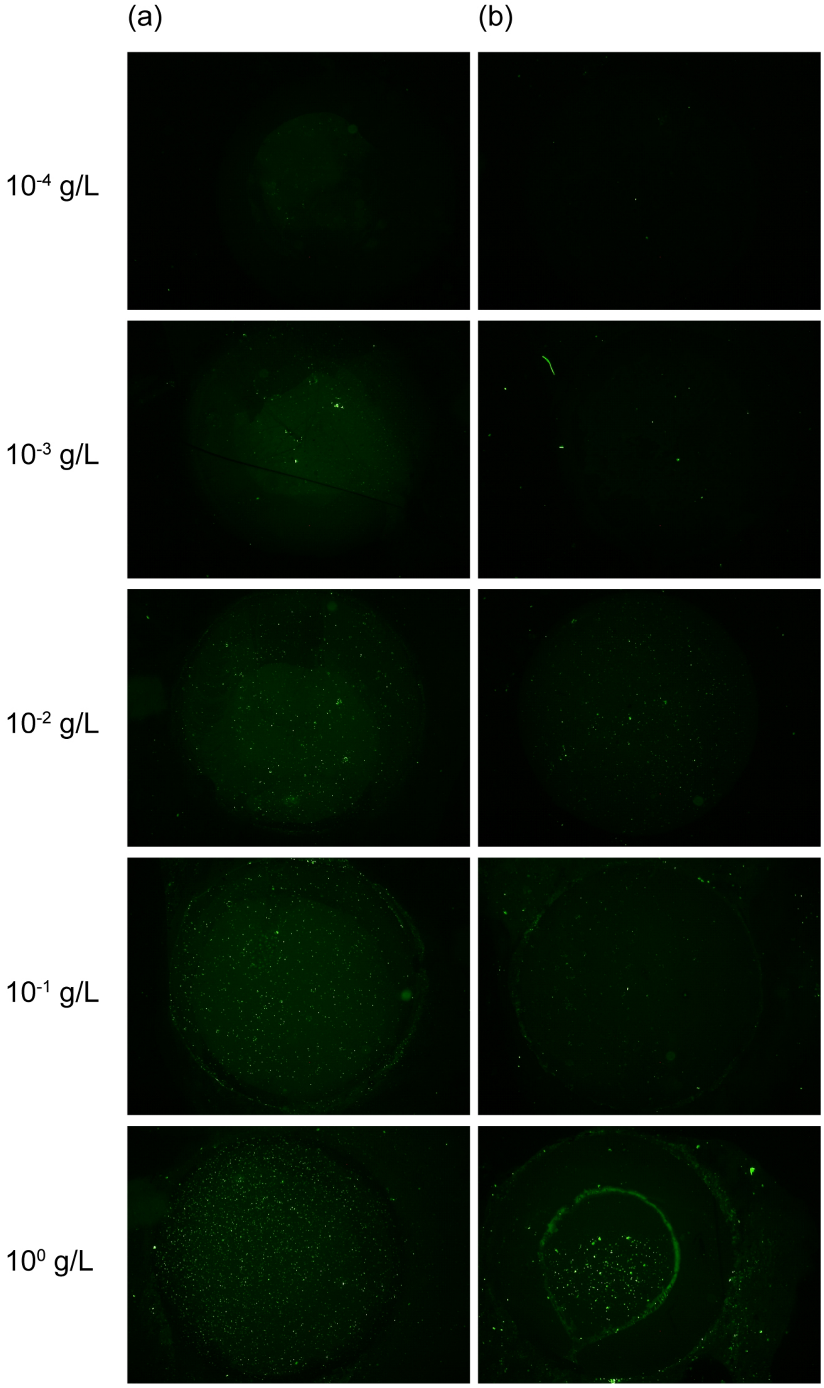

Figure S5. Breakthrough curves and amounts of protein adsorbed on (**a**) poly(acetylated Man-*t*BMA)-immobilized and (**b**) poly(Man-MA)-immobilized membranes.

(a)

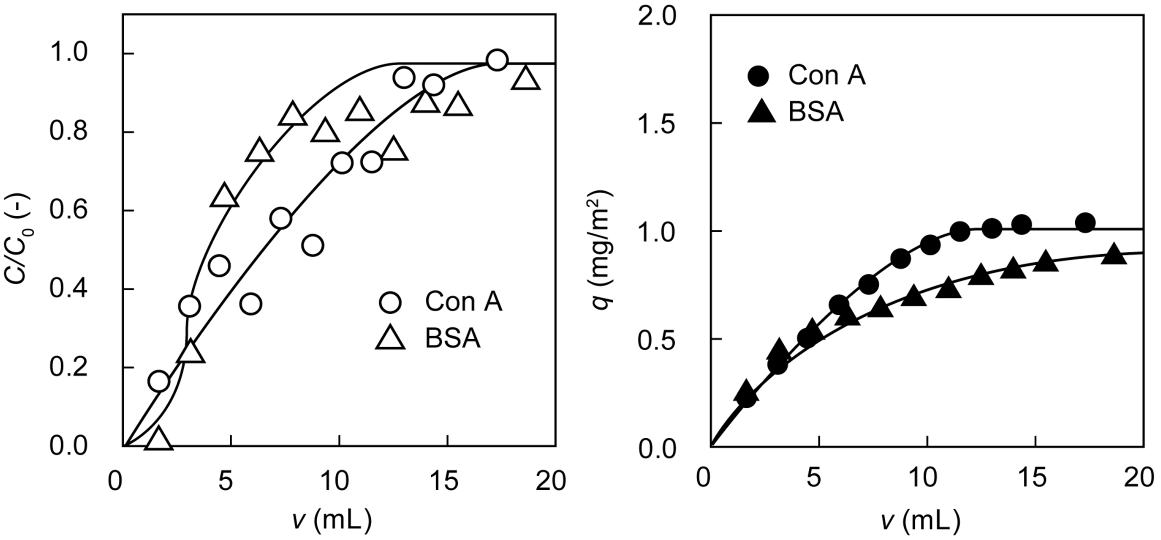

(b)

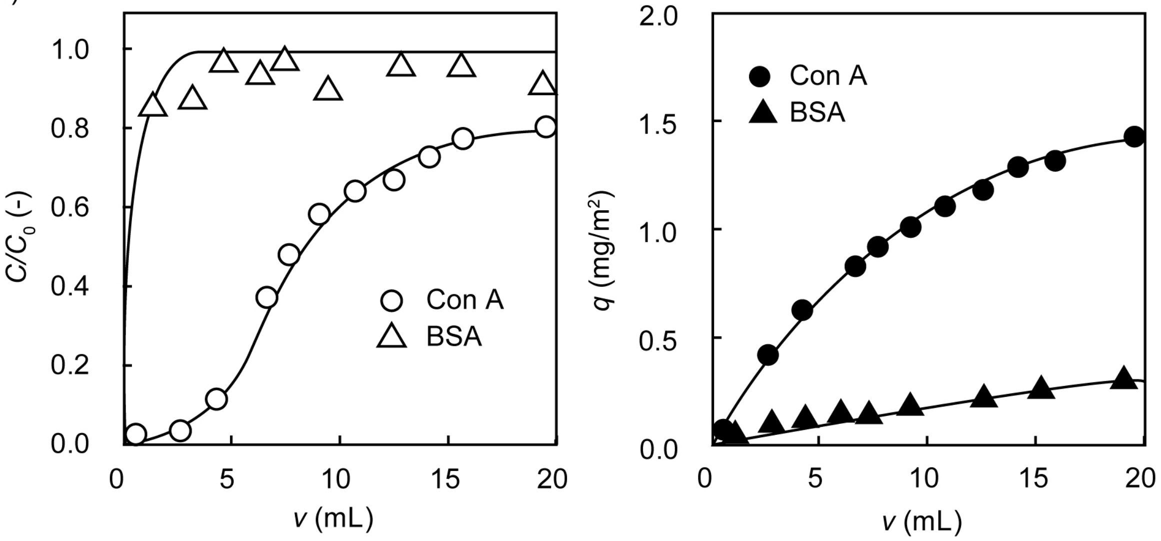

Supplement: Supplementary File 1 — Supplementary (PDF, 4025 KB) [file membranes-03-00169-s001.pdf]
